# Cardiology Beyond the Risk Factors

**DOI:** 10.5334/gh.1588

**Published:** 2026-11-13

**Authors:** Daniel José Piñeiro, Jagat Narula

**Affiliations:** 1 World Heart Federation, Argentina; 2 World Heart Federation, India

**Keywords:** Preventive cardiology, risk factors, exposome, One Health, structural determinants of health, upstream prevention

## Graphical Abstract

**Figure:**
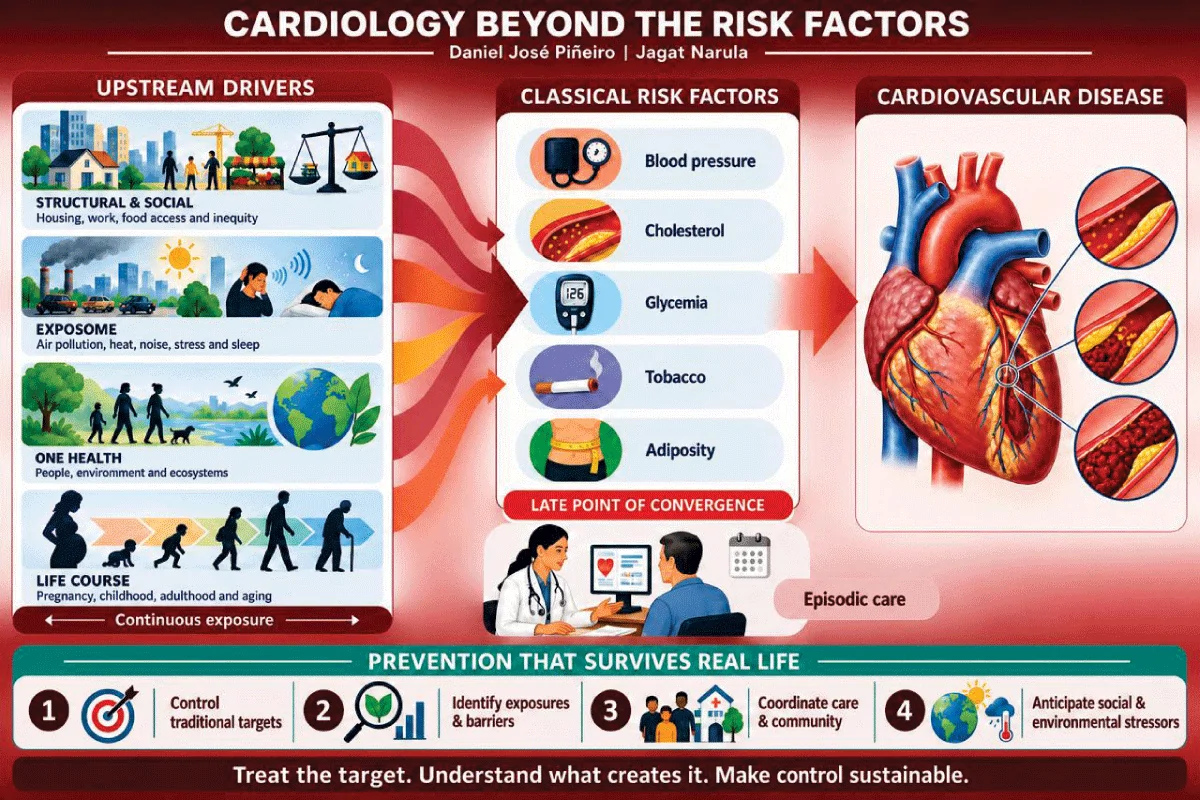


Preventive cardiology began in a single American town. When the Framingham Heart Study started following residents in 1948, cardiovascular disease was widely regarded as an inevitable companion of aging (1). By 1961, Kannel and colleagues had given the field both its organizing idea and its vocabulary: the risk factor, understood as any trait, behavior, or exposure that raises the statistical probability that an individual or a population will become ill (2). The definition carried an important caveat that is often forgotten. A risk factor is not a single, direct causal pathway; it is a marker of elevated probability (3).

That distinction matters more today than it did then. Nearly eight decades on, the risk factor remains the most productive concept in the history of chronic disease prevention. It is also increasingly the point at which prevention stops rather than starts.

Two other ideas, developed largely outside cardiology, now supply what the risk factor cannot. In 1964, veterinary and public health scholarship introduced ‘One Health,’ an integrated approach that seeks to balance and optimize the health of people, animals, and ecosystems, recognizing that the three are interdependent (4). In 2005, Wild proposed the ‘exposome’: the totality of non-genetic environmental and lifestyle exposures a person accumulates from gestation to death, offered explicitly as the complement to the genome, since genetics alone could not explain the burden of chronic disease (5). In 2009, the Canadian Heart Health Strategy and Action Plan distinguished between downstream drivers of cardiovascular disease, such as individual behaviors and biological risk factors, and the upstream social and structural conditions that shape those risks (6). Prevention aimed downstream modifies behavior and treats clinical manifestations in people already affected. Prevention aimed upstream intervenes where the problem originates.

Our argument is one. Since the 1950s, preventive cardiology has been built, with genuine success, on the control of proximal risk factors. But those factors are best understood not as the beginning of the causal story; they are its late point of convergence, the place where deeper biological, environmental, and social trajectories finally become measurable in a clinic. Taking the exposome, structural determinants, and One Health seriously does not mean abandoning traditional targets. It means understanding the conditions that generate those targets, sustaining them over time, and ultimately deciding whether their control is achievable in the real world.

## The Risk Factor as a Late Arrival

The classical approach operates on what can be measured in a consulting room: blood pressure, cholesterol, glycemia, tobacco use, and adiposity. Risk is estimated, pharmacological therapy is initiated, and behavioral change is advised. This framework is indispensable because it maps onto direct causal mechanisms; as the burden of risk factors accumulates, atherosclerotic disease not only progresses in extent but may express more vulnerable plaque phenotypes (7).

Yet even when the clinical reasoning is sound and the prescription is guideline-concordant, implementation runs into a structural asymmetry. Exposure to risk is continuous; clinical intervention is episodic. The patient’s environment does not change at the pace of the consultation.

Structural determinants: social, commercial, occupational, environmental, and contextual—precede the classical risk factors and systematically produce them. The idea of ‘moving upstream’ in cardiovascular prevention, toward the causes of the causes, has circulated in cardiovascular public health for well over a decade (8). Its clinical urgency has grown with multimorbidity, where stacking disease-specific interventions intensifies polypharmacy, treatment burden, and adverse effects without any guarantee of durability.

The upstream perspective is not abstract. It surfaces in the questions clinicians ask themselves every week. Why do some patients never reach normal blood pressure or weight targets despite repeated adjustment? Why does adherence fall away at predictable moments? Why does smoking relapse cluster around stress and material hardship? Why is sleep so reliably wrecked by shift work, noise, overcrowding, and insecurity? In most of these cases, prevention fails not for want of pharmacological efficacy, but because the implementation system in which treatment must be sustained is fragile.

## Risk Is Not Distributed at Random

Much of the value of the upstream framework lies in its capacity to explain persistent gradients. Evidence synthesized on the social determinants of cardiovascular health suggests that disadvantages such as food insecurity act on cardiovascular outcomes largely through the very behavioral domains clinicians already target: diet, physical activity, nicotine exposure, and sleep and that composite metrics such as Life’s Essential 8 may mediate a substantial share of the association between social disadvantage and cardiovascular events (9). The clinical implication is sharp: if social determinants enter pathophysiology through the same doors we are trying to close, then ignoring them is not a neutral omission. It costs explanatory and predictive power.

The same lesson appears in the feasibility literature. Population-based cardiovascular screening in primary care has shown that a single brief clinician-led visit can uncover high prevalences of hypertension, obesity, suboptimal low-density lipoprotein cholesterol, prediabetes, and smoking, with smoking, in particular, following a pronounced socioeconomic gradient (10). Effective prevention, therefore, cannot consist of recommending behaviors. It must begin from the recognition that exposure to risk environments is differential and structurally patterned.

This is where the argument becomes a global health argument rather than a national one. Gradients within countries are reproduced, amplified between them. The populations now carrying the fastest-growing share of cardiovascular burden are also those with the least capacity to absorb it, where health systems are thinnest, out-of-pocket payment is highest, food and tobacco environment is least regulated, and environmental exposures are most intense. A prevention model that presumes a stable clinic, an affordable pharmacy, and a modifiable home environment does not travel well. One that begins upstream does, because upstream instruments the price and composition of food, the regulation of tobacco and alcohol, the cleanliness of cooking and ambient air, the safety of streets, the security of work, as well as operates at a population scale and does not depend on a per-patient encounter that many health systems cannot yet guarantee. For much of the world, the upstream approach is not a refinement of preventive cardiology. It is the only version of it that can reach the people who need it most.

## The Exposome: Residual Risk, and Whether Treatment Can Survive Contact with Life

The exposome integrates the totality of exposures across the life course: chemical, physical, environmental, psychosocial, behavioral, and their interaction with biology and context. In preventive cardiology, it functions as a lens for interpreting residual risk and clinical heterogeneity. Two patients with near-identical classical profiles may diverge substantially in true risk due to differences in air pollution, heat, noise, chronic stress, sleep, access to healthy food, occupational demands, or neighborhood conditions. Adopting the exposome as a paradigm for the future of cardiovascular care has been described as a shift in perspective that permits a more holistic account of cardiovascular etiology, spanning urban planning and air quality through financial strain and dietary patterns (11).

Crucially, the exposome does not require measuring everything to be useful. It requires abandoning the assumption that what is not measured is not operative. A preventive history can readily incorporate the domains that most often explain therapeutic failure: how sleep is actually organized, working hours and shift patterns, the cost of medicines, food insecurity, safe places to be physically active, occupational exposure to heat or smoke, and caregiving stress. This information is qualitative, but it allows a clinician to stratify implementability, not merely which intervention works in theory, but which one can survive in this patient’s life.

A smaller example makes the same point about measurement. Recent evidence on adiposity distribution indicates that measures of central adiposity refine cardiovascular and mortality risk beyond body mass index (BMI) categories, identifying elevated risk among people classified as normal weight by BMI alone (12). That is precisely the exposome logic in miniature: the risk phenotype emerges from a trajectory of exposures and is rarely captured by any single marker.

## One Health: Prevention Has an Ecology

One Health is usually framed around the interdependence of human, animal, and environmental health. Its contribution to preventive cardiology is broader than zoonosis. Cardiovascular prevention takes place inside a physical and social ecosystem that either amplifies or buffers risk. The Green One Health Cardiology framework proposes integrating nature, environment, and sustainability into cardiology practice, aligning prevention with equity and resilience, and making explicit that protecting ecosystems is part of protecting cardiovascular health (13). This should not be read as an agenda external to clinical work. Heat, pollution, noise, and lack of access to green space are determinants of exposure, and therefore of risk and of decompensation.

One Health also reframes vulnerability to population-level stressors. The COVID-19 pandemic served as a systemic stress test, and the accumulated evidence indicates that better baseline cardiovascular health was associated with a lower risk of severe disease, with physical activity, BMI, blood pressure control, and sleep among the contributing factors (14). The lesson generalizes well beyond one virus: populations with stronger cardiovascular health are more resilient to external shocks, whether infectious, climatic, or economic. In an era of intersecting global crises, resilience itself is a preventive endpoint.

## Life Course: Risk Is Established Long Before It Is Measured

The upstream view acquires a further dimension across the life course. Early exposures shape cardiometabolic risk and may carry intergenerational consequences. Cohort data have linked poorer maternal cardiovascular health during pregnancy, assessed with composite metrics, to a higher risk of developmental delay in the offspring, suggesting that cardiovascular health in gestation behaves as an integrated indicator of multiple exposures and behaviors (15). The outcome there is not itself cardiovascular, which is exactly what makes the point: the window in which risk is established opens far earlier than the moment we first measure low-density lipoprotein cholesterol or diagnose hypertension.

Non-classical markers of trajectory point the same way. Premature menopause, for instance, has been associated with a higher subsequent risk of hypertension, indicating a need for earlier surveillance in defined subgroups (16). Such biographical and reproductive markers make little sense within a narrow list of standard risk factors. They make immediate sense within a framework of trajectories and susceptibility.

## What This Asks of Us

Adopting the language of upstream drivers, the *exposome*, and *One Health* does not mean converting the clinical encounter into a comprehensive social intervention. It means accepting that the success of prevention depends as much on the sustainability of therapy as on its selection. In practice, this points to a preventive cardiology that combines four things: rigorous control of traditional targets; deliberate identification of the exposures and barriers that govern adherence, sleep, diet, and physical activity; coordination with primary care, nursing, rehabilitation, clinical pharmacy, and community resources; and anticipation of the environmental and social stressors that precipitate decompensation.

It also asks something about how we generate and grade evidence. If implementability is part of effectiveness, then trials, registries, and guidelines need to report the contexts in which interventions were delivered and sustained, not only the magnitude of effect under favorable conditions. A therapy that works everywhere it is studied, but nowhere it is needed, has not been fully evaluated.

At the level of systems and policy, the same logic argues for aligning cardiovascular disease prevention with food, housing, labor, transport, air quality, and climate policy, the instruments that actually set the level of exposure for whole populations, and that determine whether the clinical encounter is working with the environment or against it.

Shifting from classical risk factors to upstream drivers is not a matter of adding complexity for academic interest. It is a matter of repositioning prevention at the point of origin and of perpetuating risk. In the era of the exposome and One Health, preventive cardiology will be defined less by the enumeration of targets than by the capacity to sustain, in real-world conditions and across unequal ones, the continuous control of those targets, reducing inequity and building clinical and population resilience as it goes.
